# Serum amyloid A–containing HDL binds adipocyte-derived versican and macrophage-derived biglycan, reducing its antiinflammatory properties

**DOI:** 10.1172/jci.insight.142635

**Published:** 2020-09-24

**Authors:** Chang Yeop Han, Inkyung Kang, Mohamed Omer, Shari Wang, Tomasz Wietecha, Thomas N. Wight, Alan Chait

**Affiliations:** 1 Division of Metabolism, Endocrinology, and Nutrition, Department of Medicine, University of Washington, Seattle, Washington, USA.; 2 Matrix Biology Program, Benaroya Research Institute, Seattle, Washington, USA.; 3 Division of Cardiology, Department of Medicine, University of Washington, Seattle, Washington, USA.

**Keywords:** Cell Biology, Inflammation, Adipose tissue, Lipoproteins, Macrophages

## Abstract

The ability of HDL to inhibit inflammation in adipocytes and adipose tissue is reduced when HDL contains serum amyloid A (SAA) that is trapped by proteoglycans at the adipocyte surface. Because we recently found that the major extracellular matrix proteoglycan produced by hypertrophic adipocytes is versican, whereas activated adipose tissue macrophages produce mainly biglycan, we further investigated the role of proteoglycans in determining the antiinflammatory properties of HDL. The distributions of versican, biglycan, apolipoprotein A1 (the major apolipoprotein of HDL), and SAA were similar in adipose tissue from obese mice and obese human subjects. Colocalization of SAA-enriched HDL with versican and biglycan at the cell surface of adipocyte and peritoneal macrophages, respectively, was blocked by silencing these proteoglycans, which also restored the antiinflammatory property of SAA-enriched HDL despite the presence of SAA. Similar to adipocytes, normal HDL exerted its antiinflammatory function in macrophages by reducing lipid rafts, reactive oxygen species generation, and translocation of Toll-like receptor 4 and NADPH oxidase 2 into lipid rafts, effects that were not observed with SAA-enriched HDL. These findings imply that SAA present in HDL can be trapped by adipocyte-derived versican and macrophage-derived biglycan, thereby blunting HDL’s antiinflammatory properties.

## Abstract 2

Versican in adiopcytes and biglycan in macrophages trap serum amyloid A-containing HDL, thereby blocking HDL’s anti-inflammatory properties.

## Graphical abstract

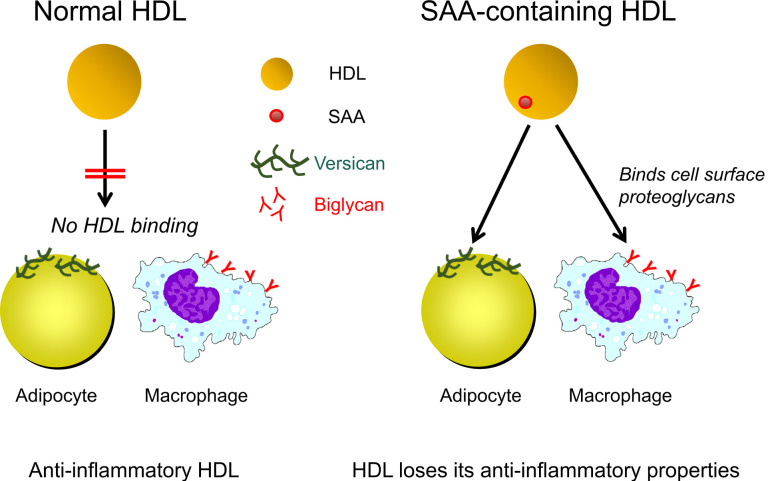

## Introduction

Chronic caloric excess from glucose or fatty acids leads to obesity, which is characterized by accumulation of macrophages in adipose tissue in response to chemotactic factors generated by adipocytes (1–3). Both adipocytes and macrophages secrete a number of proinflammatory molecules, which may lead to chronic low-grade inflammation and insulin resistance in obesity (4–6). One of these inflammatory molecules is serum amyloid A (SAA), which is chronically and modestly elevated in obese mice (7–10) and humans (11–14). Moreover, specific components of extracellular matrix molecules (ECM) also increase during the development of obesity, albeit their roles are incompletely understood. Some ECM molecules serve as a scaffold at the cell surface that can bind and sequester chemokines and proinflammatory molecules. One class of ECM molecules that has immunoregulatory properties and that has received little attention in adipose tissue is proteoglycans.

In addition to its role in reverse cholesterol transport, HDL has antiinflammatory properties in various cell types. We previously have shown that HDL from healthy chow-fed mice and healthy human subjects reduces inflammatory responses in adipocytes (15). Moreover, adipose tissue inflammation is blunted in mice overexpressing apolipoprotein A1 (APOA1), the major apolipoprotein of HDL in humans (15). We also have shown that HDL loses its antiinflammatory effect on adipocytes when it acquires the proinflammatory molecule SAA because of entrapment of SAA-enriched HDL (also called SAA-HDL) by proteoglycans at the cell surface of hypertrophic adipocytes, thereby blocking access of HDL to the adipocyte plasma membrane and inhibiting its normal function (16). The nature of the proteoglycan that accounted for this interaction was unknown until our identification of this molecule as versican, which is the major proteoglycan produced by hypertrophic adipocytes (17). We also showed that adipose tissue macrophages mainly synthesize and secrete biglycan, a proteoglycan found in the ECM in obesity (17). SAA-containing HDL physically binds isolated biglycan (18). To further understand the role of these particular proteoglycans in their interaction with HDL, we studied their role in regulating inflammation in both adipocytes and macrophages in vitro and in adipose tissue from obese mice and humans. We now demonstrate that versican and biglycan colocalize with both APOA1, HDL’s major apolipoprotein, and SAA in adipose tissues of mice challenged with a high-fat, high-sucrose (HFHS) diet and in obese subjects undergoing gastric bypass surgery. Moreover, versican at the cell surface of hypertrophic adipocytes traps SAA-enriched HDL, leading to impairment of HDL’s antiinflammatory function in vitro. Silencing versican expression in adipocytes restored the antiinflammatory function of HDL even if the lipoprotein was enriched in SAA. Similar results were observed in macrophages, in which biglycan at the cell surface of thioglycollate-elicited (TG-elicited) peritoneal macrophages associated with SAA-enriched HDL, leading to impairment of HDL’s antiinflammatory function in the macrophages. Silencing biglycan restored the antiinflammatory function of HDL in macrophages even if the lipoprotein was enriched in SAA. Similar to adipocytes (16), normal HDL exerts its antiinflammatory function in macrophages by reducing lipid rafts (LRs), reactive oxygen species (ROS) generation, and translocation of Toll-like receptor (TLR) 4 and NADPH oxidase (NOX) 2 into LRs, while these effects were not observed with SAA-enriched HDL.

Collectively, our results suggest that adipocyte-derived versican and macrophage-derived biglycan function as a scaffold that, by trapping SAA when present in HDL, will reduce the accessibility of HDL to the plasma membrane of both these cell types, thereby blunting its antiinflammatory properties.

## Results

### Versican, biglycan, APOA1, and SAA are similarly distributed in adipose tissue from obese mice and human subjects.

**Figure 1:**
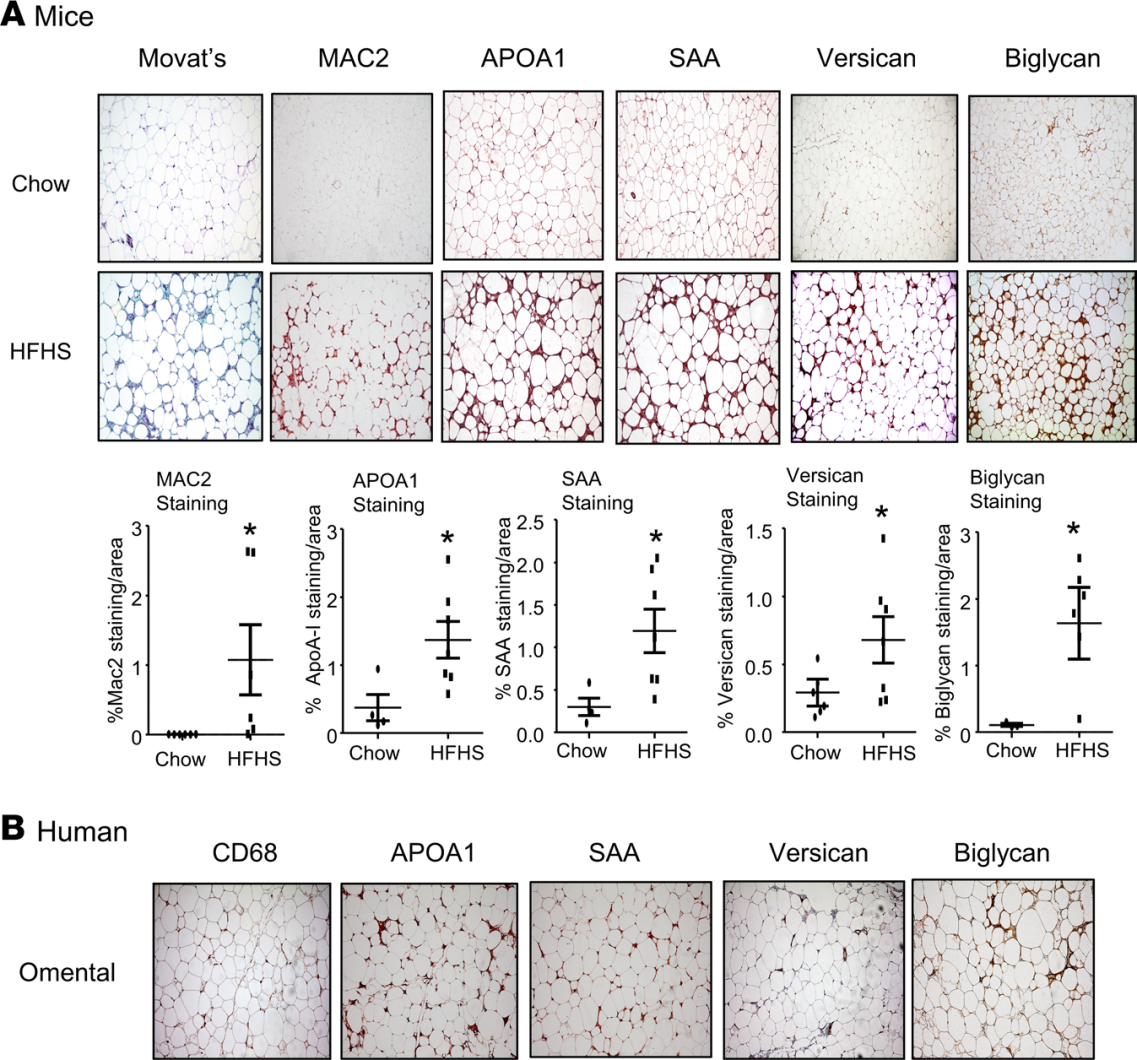
Immunohistochemical staining of versican and biglycan shows a similar distribution with the staining of SAA and APOA1 in the epididymal white adipose tissue from mice fed an HFHS diet and in the omental fat tissue from human obese subjects undergoing gastric bypass surgery. (**A**) Mice were fed a chow or HFHS diet for 16 weeks, after which adipose tissue was obtained and immunostained with the antibodies shown. Tissues were photographed using microscopy (original magnification, ×60). Quantitation of the immunostaining is shown in the lower panel (*n* = 5–7, mean ± SEM). *P < 0.001 vs. chow. (**B**) Omental fat was obtained from gastric bypass patients and immunostained with the antibodies shown. Tissues were photographed using microscopy (original magnification, ×60). The pictures of MAC2, CD68, versican, and biglycan staining are from our previous publication (17); APOA1 and SAA staining were performed on adjacent sections.

Previously, we showed that SAA-enriched HDL colocalized with a chondroitin sulfate–containing proteoglycan in adipose tissue from mice fed an HFHS diet for 16 weeks but not in lean mice (16). We subsequently have shown that versican derived from adipocytes and biglycan derived from adipose tissue macrophages were increased in obesity (17). To further understand the role of these proteoglycans in the retention of SAA-enriched HDL, immunostaining for APOA1 and SAA was performed on adjacent sections of adipose tissues from our previous study (17), in which C57BL/6 mice were made obese by feeding an HFHS diet for 16 weeks, and adjacent sections of human omental adipose tissue from gastric bypass patients. In epididymal adipose tissue from obese mice, APOA1 and SAA staining showed a similar distribution to versican and biglycan staining (Figure 1A). Similar results were observed in omental fat from human subjects (Figure 1B).

### SAA-enriched HDL associates with adipocyte-derived versican and loses its antiinflammatory properties in adipocytes.

**Figure 2:**
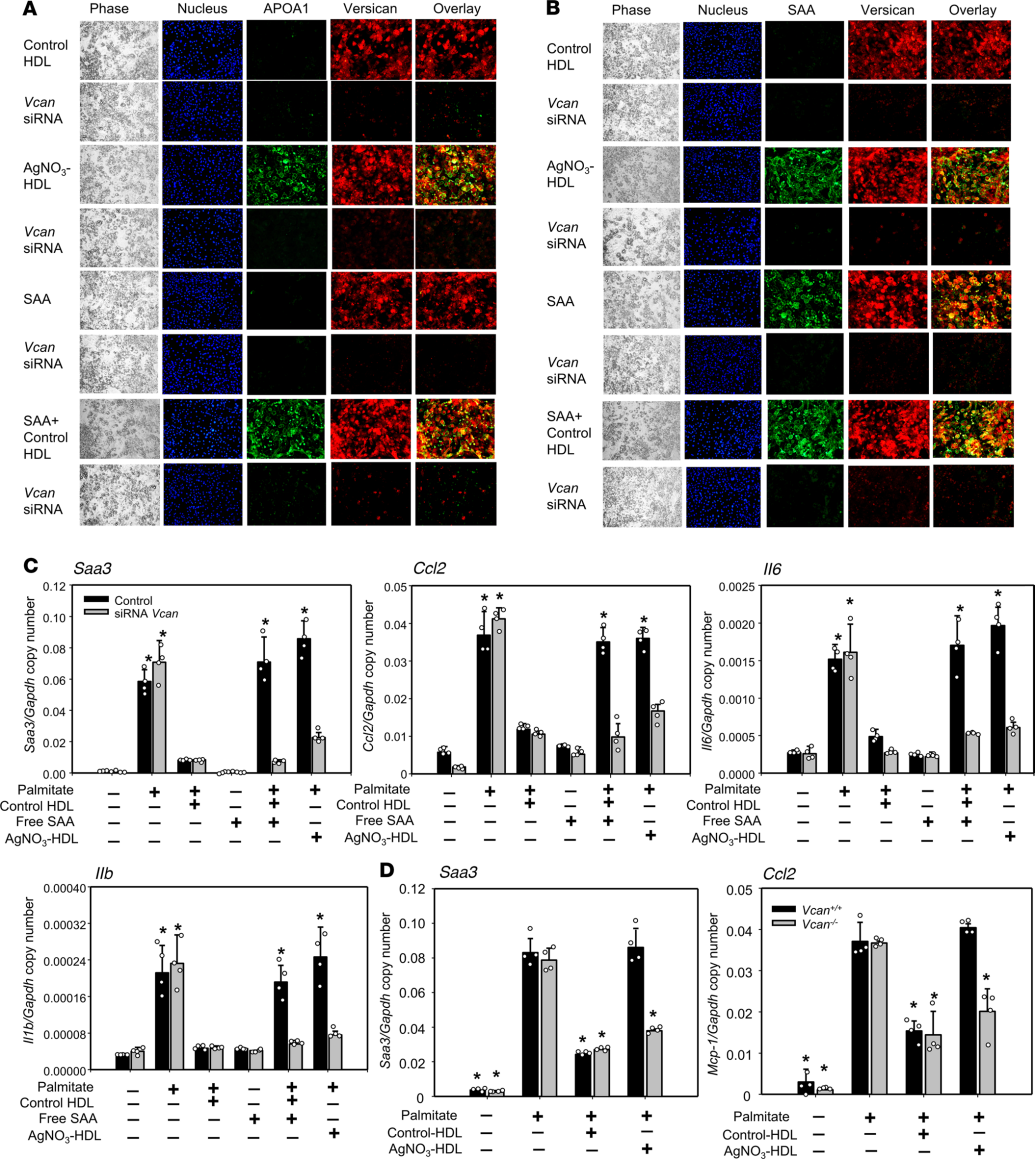
Versican colocalizes with HDL isolated from AgNO_3_-injected mice at the cell surface of 3T3-L1 adipocytes. Free SAA and HDL from PBS- or AgNO_3_-injected C57BL/6 mice were isolated. Some adipocytes were transfected with siRNA specific for versican (*Vcan*) for 3 days. After exposure to free SAA (15 μg/mL) and/or these HDL preparations (50 μg protein/mL) for 6 hours, 3T3-L1 adipocytes were fixed in 2% formalin for 5 minutes. After extensively washing, (**A**) APOA1 and versican were stained using anti-versican (shown in red) and anti-APOA1 (shown in green) antibodies, or (**B**) SAA and versican were stained using anti-versican (shown in red) and anti-SAA (shown in green) antibodies and photographed by fluorescence microscopy (Nikon Eclipse 80i, original magnification, ×200). Cell nuclei were counterstained with DAPI (blue). Cell morphology was shown by phase-contrast photography (left). Merged fluorescence (overlay) is shown in yellow. (**C**) Some adipocytes were transfected with siRNA specific for versican (*Vcan*) for 3 days, or (**D**) preadipocytes from adipocyte-specific *Vcan*-deficient (*Vcan*^–/–^) or WT control (*Vcan^+/+^*) mice were differentiated into adipocytes. After that, HDL (50 μg protein/mL) was added for 6 hours. After extensive washing, the adipocytes were incubated with or without palmitate (250 μmol/L) for 24 hours before measurement of *Saa3*, *Ccl2*, *Il1b*, and *Il6* gene expression. Data are representative of 3 independent experiments (*n* = 4) with mean ± SEM. *P < 0.001 vs. control HDL. ANOVA and Bonferroni’s post hoc test.

When the ECM of adipocytes was digested by chondroitin ABC lyase, the antiinflammatory properties of SAA-enriched HDL were restored (16). We subsequently identified the chondroitin sulfate–rich proteoglycan produced by hypertrophic adipocytes as versican (17), suggesting that it was responsible for mediating the interaction with SAA in HDL from inflamed subjects. To determine whether SAA-enriched HDL colocalizes with versican at the cell surface of 3T3-L1 adipocytes, the cells were immunostained with antibodies against APOA1, SAA, and versican after incubation with HDL isolated from control lean, chow-fed mice or SAA-enriched HDL isolated from mice injected with AgNO_3_ to induce sterile inflammation. APOA1 and SAA (green fluorescence, Figure 2, A and B, third row) colocalized with versican (red fluorescence, Figure 2, A and B, third row) and merged (yellow fluorescence) when cells were incubated with HDL from AgNO_3_-injected mice. Conversely, when cells were incubated with control HDL, only versican staining was observed (Figure 2, A and B, first row). When versican was silenced with a specific siRNA, no APOA1, SAA, or versican staining was observed after incubation with SAA-enriched HDL (Figure 2, A and B, fourth row). This suggests that SAA-enriched HDL can be trapped by versican at the adipocyte cell surface.

To investigate whether free SAA could bind versican, purified mouse SAA (a gift from Frederick C. de Beer from University of Kentucky, Lexington, Kentucky, USA) (19) was added to 3T3-L1 adipocytes. Free SAA colocalized and merged with versican (Figure 2B, fifth row), and SAA staining disappeared following versican silencing (Figure 2B, sixth row). This suggests that SAA may bind versican even when not lipoprotein associated. Because we have shown that the presence of SAA facilitates the trapping of HDL to proteoglycans on the cell surface of adipocytes, we tested whether mixing purified free SAA with control HDL would result in colocalization of the resultant HDL particle with versican. When SAA and control HDL were coincubated with 3T3-L1 adipocytes, APOA1, SAA, and versican colocalized with each other (Figure 2, A and B, seventh row). However, this colocalization was not observed following versican silencing (Figure 2, A and B, eighth row), supporting that SAA is a critical component that permits HDL binding to versican and can be incorporated into control HDL, presumably in part by displacing APOA1 (20).

Silencing versican with a specific siRNA also restored the ability of SAA-enriched HDL from AgNO_3_-injected mice to inhibit palmitate-induced expression in adipocytes of the chemokines *Ccl2* and *Saa3*, an isotype of SAA made by extrahepatic tissues, and the cytokines *Il1b* and *Il6* (Figure 2C). Interestingly, although free SAA itself did not affect adipocyte inflammation, when SAA was incubated with control HDL from lean, chow-fed mice, the HDL lost its antiinflammatory properties (Figure 2C), again consistent with uptake of SAA by control HDL, presumably in part by displacement (20), thereby rendering the HDL dysfunctional. Moreover, when we used primary adipocytes that were differentiated from preadipocytes from adipocyte-specific *Vcan*-deficient (*Vcan^–/–^*) mice (17), SAA-containing HDL from AgNO_3_-injected mice was able to inhibit palmitate-induced expression of *Ccl2* and *Saa3*, similar to control HDL (Figure 2D). Collectively, these results indicate that versican at the cell surface of adipocytes plays an important role in trapping SAA when present in HDL, thereby blunting its antiinflammatory properties.

### HDL from mice fed a chow diet (control HDL) inhibits inflammation in peritoneal macrophages.

**Figure 3:**
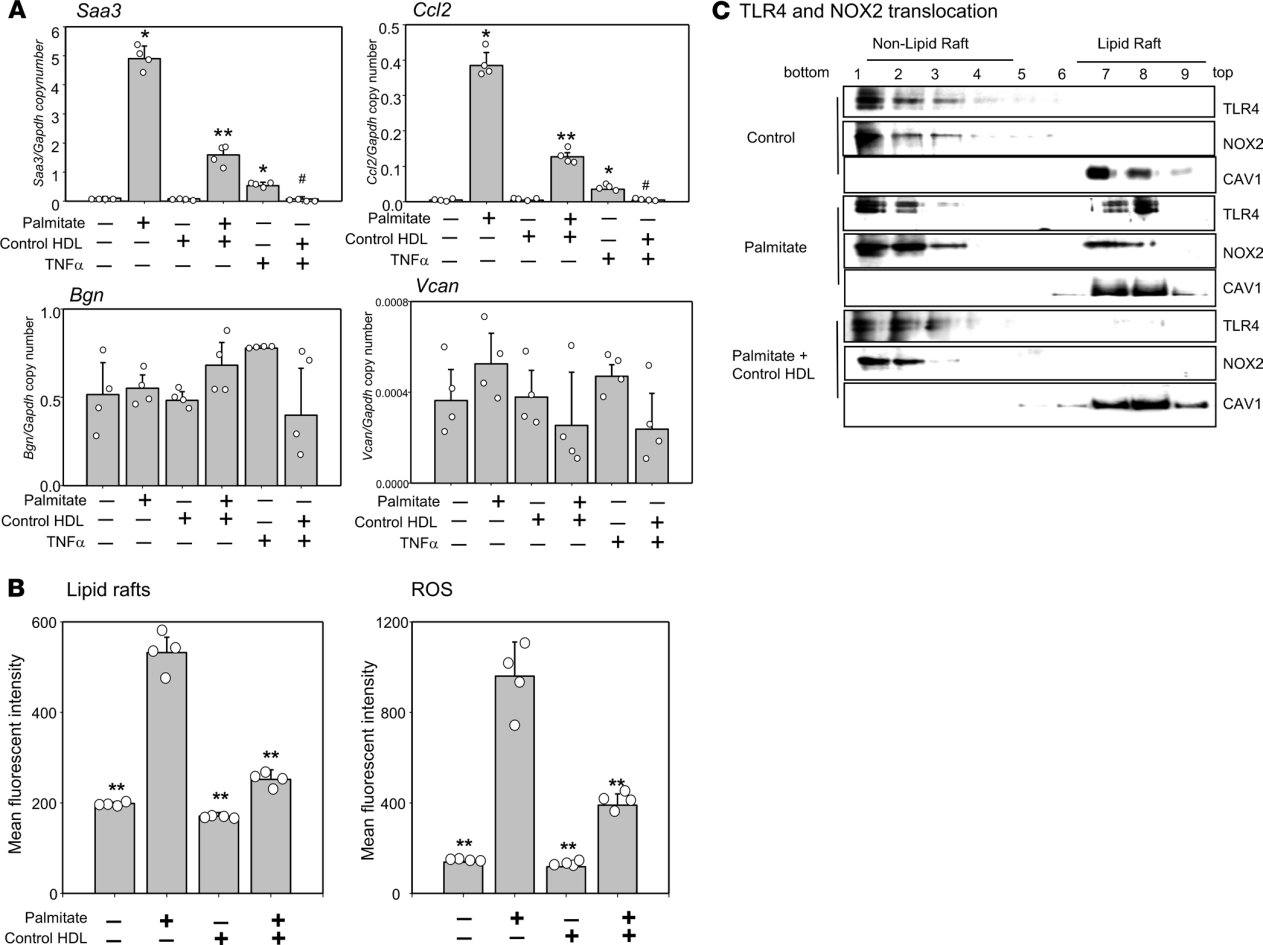
Control HDL inhibits palmitate-induced inflammation in TG-elicited peritoneal macrophages. TG-elicited peritoneal macrophages were preexposed to HDL (50 μg protein/mL) for 6 hours, after which the HDL was removed, the cells were washed, and the macrophages were incubated with or without palmitate (50 μmol/L) or TNF-α (10 ng/mL) for 24 hours before measurement of *Saa3*, *Ccl2*, *Bgn*, and *Vcan* gene expression (**A**), LR content and ROS generation (**B**), and TLR4 and NOX2 translocation to LRs (**C**), as described in Methods. Results are plotted as the mean fluorescence intensity of each sample on the vertical axis (**B**). An antibody against caveolin-1 (CAV1) was used to stain LRs (**C**). Fractions 7 to 9 contain LRs and fractions 1 to 4 are non–LR-containing fractions. Data are representative of 3 independent experiments (*n* = 4) with mean ± SEM. *P < 0.01 vs. control HDL. **P < 0.01 vs. palmitate. ^#^*P* < 0.01 vs. TNF-α. ANOVA and Bonferroni’s post hoc test.

Although it has been reported that control HDL isolated from healthy humans and mice fed a chow diet has antiinflammatory effects on macrophages (21–23), a recent study showed that control HDL had proinflammatory effects (24). Therefore, we first tested whether control HDL exhibits anti- or proinflammatory effects on TG-elicited peritoneal macrophages. Palmitate, and to a lesser extent TNF-α, treatment increased *Saa3* and *Ccl2* chemokine gene expression, which was significantly reduced by control HDL from C57BL/6 mice (Figure 3A). However, biglycan, which was highly expressed in TG-elicited peritoneal macrophages, and versican, which was expressed at low levels, were not affected (Figure 3A). We also investigated whether control HDL regulated LR content, ROS generation, and translocation of TLR4 and NOX2 protein into LRs in the plasma membrane of macrophages, similar to what we had previously observed with adipocytes (16, 25). Exposure of TG-elicited peritoneal macrophages to palmitate increased LR content and generated ROS, effects that were inhibited by preexposure to control HDL (Figure 3B). Similar to what we had previously observed with adipocytes (16), control HDL blunted palmitate-induced translocation of TLR4 and NOX2 into LRs in TG-elicited peritoneal macrophages (Figure 3C). These results suggest that the antiinflammatory effect of HDL on macrophages occurs by reducing the LR content of the plasma membrane, followed by decreased translocation of TLR4 and NOX2, which play a role in the propensity of these cells to inflammation.

### SAA-enriched HDL isolated from AgNO_3_-injected mice loses its antiinflammatory effect on peritoneal macrophages.

**Figure 4:**
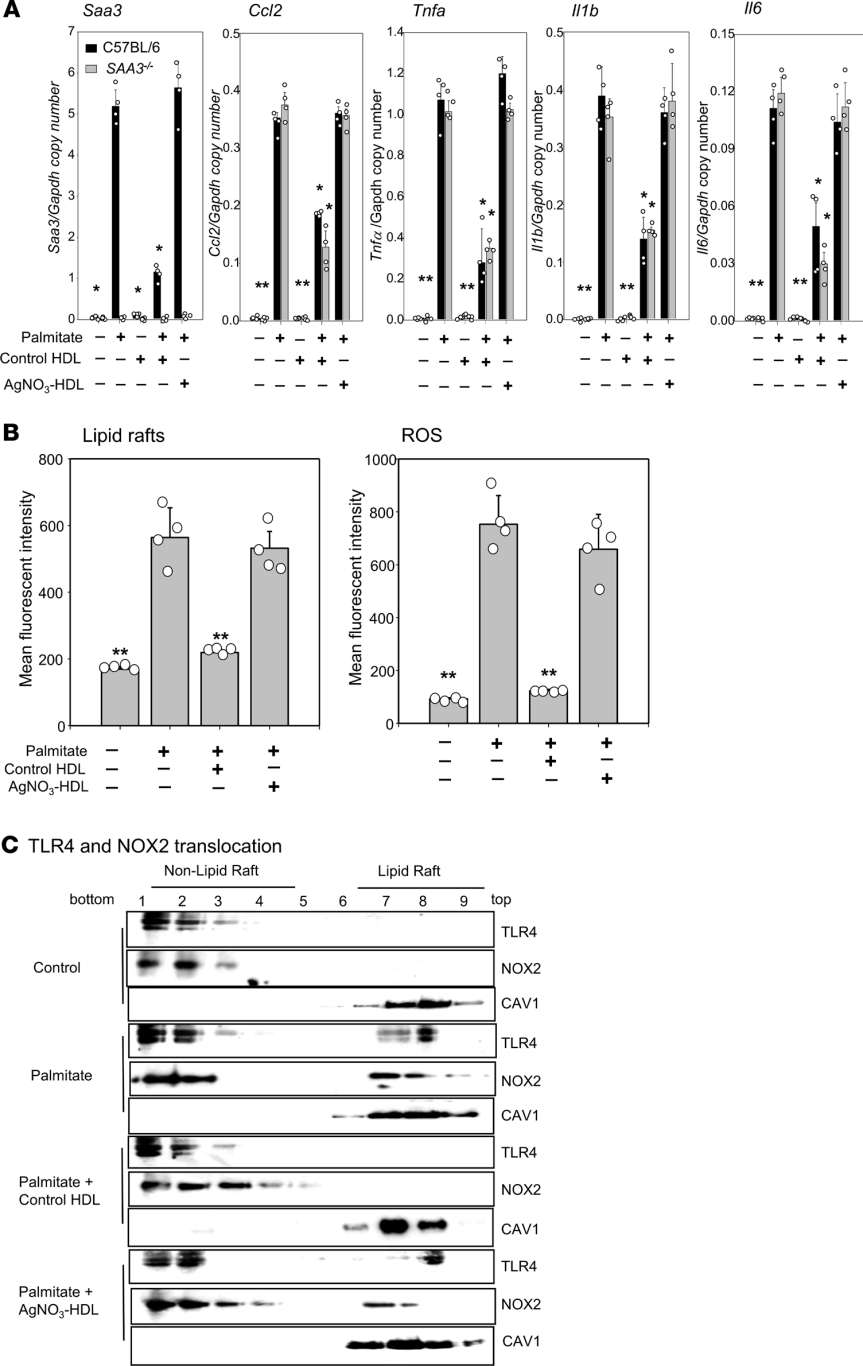
HDL from AgNO_3_-injected mice loses its ability to inhibit palmitate-induced macrophage inflammation, to suppress LR content and ROS generation, and to block the translocation of TLR4 and NOX2 into LRs in peritoneal macrophages. HDL was isolated from the plasma of AgNO_3_- or PBS-injected C57BL/6 mice. TG-elicited peritoneal macrophages isolated from control C57BL/6 (**A**–**C**) or *Saa3^–/–^* (**A**) mice were preexposed to HDL (50 μg protein/mL) for 6 hours, after which the HDL was removed, the cells were washed, and the peritoneal macrophages were incubated with or without palmitate (50 μmol/L) for 24 hours before measurement of *Saa3*, *Ccl2*, *Tnfa*, *Il1b*, and *Il6* gene expression (**A**); LR content and ROS generation (**B**); and TLR4 and NOX2 translocation to LRs (**C**). Data are representative of 3 independent experiments (*n* = 4) with mean ± SEM. *P < 0.001 vs. control HDL. **P < 0.001 vs. palmitate. ANOVA and Bonferroni’s post hoc test.

To determine whether HDL from AgNO_3_-injected mice loses its antiinflammatory function on macrophages as it does on adipocytes (16), TG-elicited peritoneal macrophages were preexposed to control HDL or HDL isolated from AgNO_3_-injected mice before stimulation with palmitate. While control HDL blocked *Saa3*, *Ccl2*, *Tnfa*, *Il1b*, and *Il6* gene expression, HDL isolated from AgNO_3_-injected mice failed to inhibit the palmitate-induced expression of these genes (Figure 4A). The extrahepatic form of SAA, SAA3, can be produced by macrophages (26) and can be incorporated into HDL particles after the injection of large doses of lipopolysaccharide (LPS) (27, 28) and to a minor degree after the injection of AgNO_3_ (28). Because endogenous SAA3 produced by macrophages could potentially affect HDL function, we compared peritoneal macrophages isolated from WT control C57BL/6 mice and SAA3-deficient (*Saa3^–/–^*) mice. Both control HDL and HDL isolated from AgNO_3_-injected mice showed the same effect on peritoneal macrophages from both WT and *Saa3^–/–^* mice (Figure 4A), indicating that endogenous production of SAA3 by peritoneal macrophages does not affect the function of control HDL and SAA-HDL. To determine whether other macrophages respond to control HDL and SAA-containing HDL in a similar manner as peritoneal macrophages, bone marrow–derived macrophages (BMDMs) were preexposed to control and SAA-enriched HDL before differentiation into M1 or M2 phenotypes using standard methodology. Control HDL blunted the expression of the M1 macrophage markers *Nos2* and *Tnfa* in macrophages after exposure to interferon-γ and LPS (Supplemental Figure 1; supplemental material available online with this article; https://doi.org/10.1172/jci.insight.142635DS1). Preexposure to control HDL also blunted the expression of the M2 markers, *Arg1* and *Cd206*, after exposure of BMDMs to IL-4 (Supplemental Figure 1). Conversely, HDL isolated from AgNO_3_-injected mice did not reduce the differentiation of BMDMs into either M1- or M2-type macrophages (Supplemental Figure 1).

In contrast to control HDL (Figure 3), HDL isolated from AgNO_3_-injected mice did not inhibit the increase in plasma membrane LR content and generation of ROS induced by palmitate (Figure 4B) or block translocation of TLR4 and NOX2 into LRs in macrophages (Figure 4C), similar to what we observed previously in adipocytes with respect to LR content, TLR4 and NOX4 translocation to LRs, and ROS generation (16).

### SAA-enriched HDL loses its antiinflammatory function by binding biglycan at the surface of macrophages.

**Figure 5:**
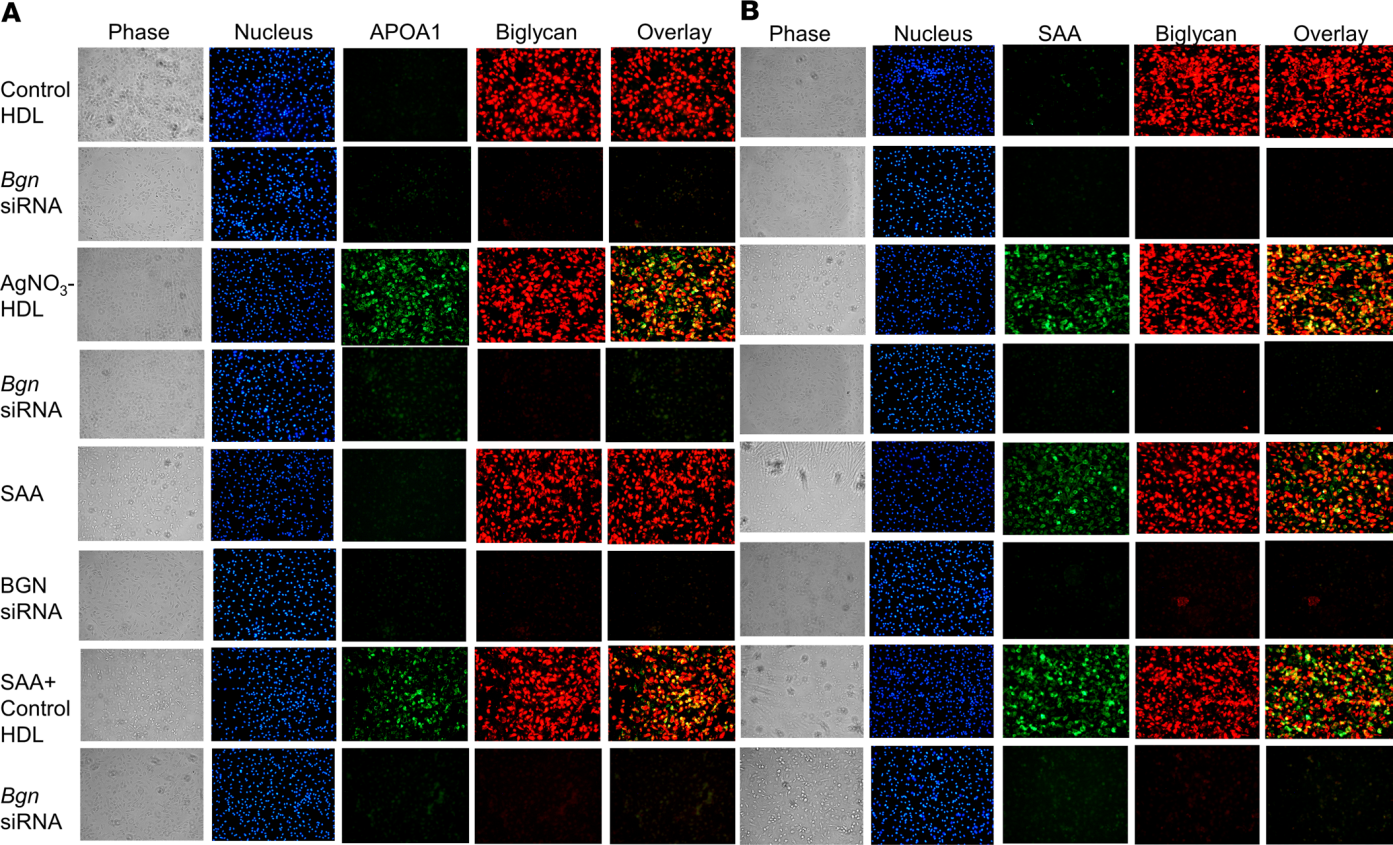
HDL isolated from AgNO_3_-injected mice colocalizes with biglycan at the cell surface of peritoneal macrophages. HDL from PBS- and AgNO_3_-injected C57BL/6 mice was isolated. After exposure to these HDL preparations (50 μg protein/mL) for 6 hours, TG-elicited peritoneal macrophages from *Saa3^–/–^* were fixed in 2% formalin for 5 minutes (**A** and **B**). After extensive washing, (**A**) APOA1 and biglycan were stained using anti-biglycan (red) and anti-APOA1 (green) antibodies, or (**B**) SAA and biglycan were stained using anti-biglycan (red) and ant-SAA (green) antibodies and photographed by fluorescence microscopy (Nikon Eclipse 80i, original magnification, ×200).

Since SAA-HDL binds purified biglycan through an interaction between SAA and biglycan (18) and since biglycan is the major proteoglycan produced by macrophages (17), we performed experiments to determine whether biglycan was responsible for the loss of antiinflammatory properties on macrophages of SAA-HDL similar to what we have shown with versican in adipocytes. After incubation of SAA-enriched HDL from AgNO_3_-injected mice or control HDL with TG-elicited peritoneal macrophages, the cells were stained with antibodies against APOA1, SAA, and biglycan. To exclude the possibility that SAA3 endogenously expressed in peritoneal macrophages could interfere with the staining of SAA in HDL, TG-elicited peritoneal macrophages from *Saa3^–/–^* mice were used. APOA1 and SAA (green fluorescence, Figure 5, A and B, third row) colocalized with biglycan (red fluorescence, Figure 5, A and B, third row) and merged (yellow fluorescence) when cells were incubated with HDL from AgNO_3_-injected mice. Conversely, when cells were incubated with control HDL, only biglycan staining was observed (Figure 5, A and B, first row). The specific role of this proteoglycan was confirmed by silencing biglycan in TG-elicited peritoneal macrophages with a specific siRNA, after which no APOA1, SAA, or biglycan staining was observed following incubation with SAA-enriched HDL (Figure 5, A and B, fourth row). These findings suggest that SAA-enriched HDL can be trapped by biglycan at the cell surface of macrophages, similar to the trapping of SAA-enriched HDL at the surface of adipocytes by versican.

To investigate whether free SAA could bind to biglycan, purified SAA was added to TG-elicited peritoneal macrophages and shown to colocalize and merge with biglycan (Figure 5B, fifth row). Following biglycan silencing in peritoneal macrophages, SAA staining was not observed (Figure 5B, sixth row), suggesting that SAA itself can bind to biglycan at the surface of macrophages. Moreover, when free SAA and control HDL were coincubated with TG-elicited peritoneal macrophages, APOA1, SAA, and biglycan colocalized with each other (Figure 5, A and B, seventh row), an effect that was not observed after biglycan silencing (Figure 5, A and B, eighth row). These findings strongly suggest the SAA component of SAA-enriched HDL is responsible for biglycan binding at the surface of macrophages.

**Figure 6:**
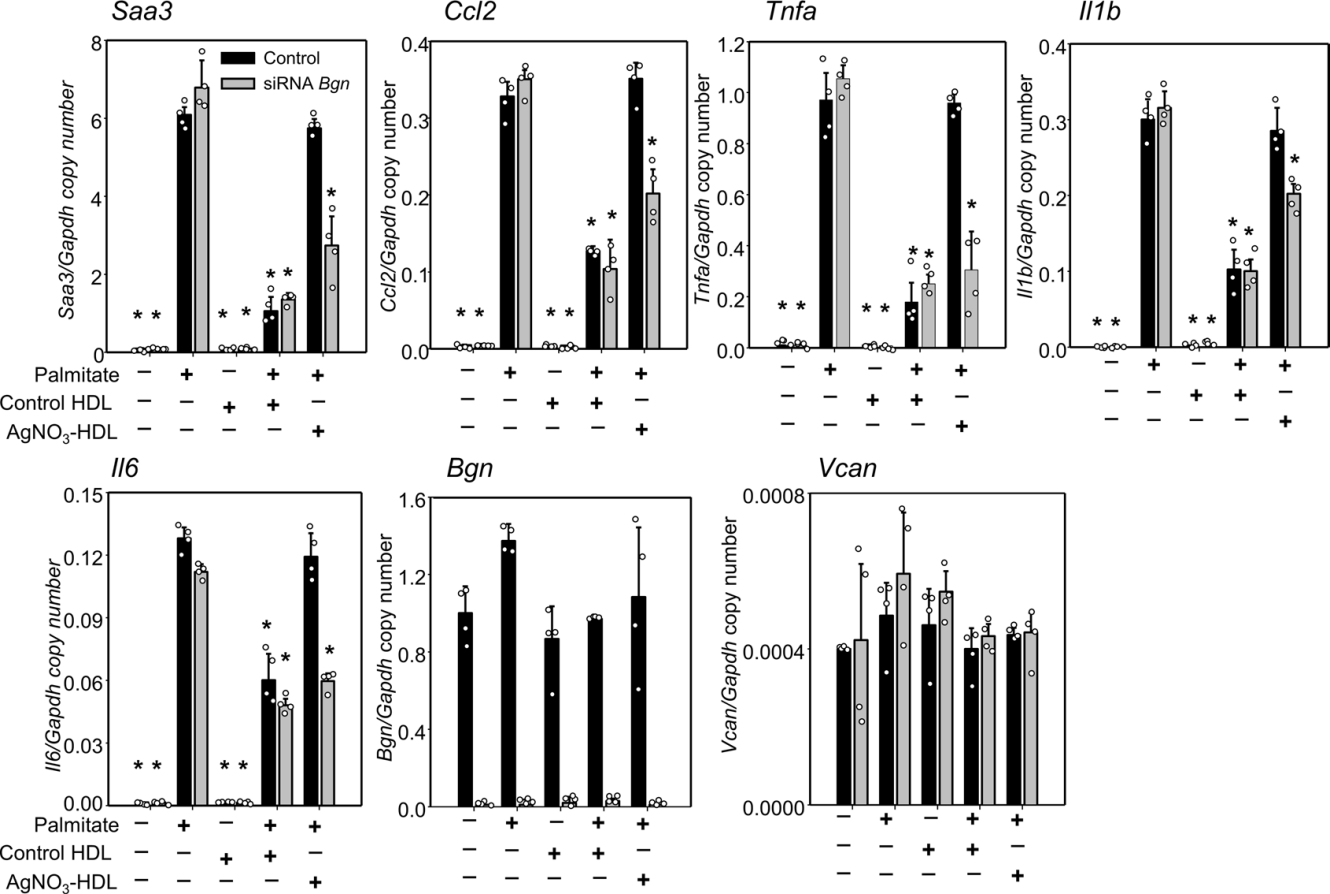
Silencing of biglycan restores antiinflammatory effect of HDL isolated from AgNO_3_-injected mice on peritoneal macrophages. HDL from PBS- and AgNO_3_-injected C57BL/6 mice was isolated. Some peritoneal macrophages were transfected with a siRNA specific for biglycan (*Bgn*) for 3 days. After that, HDL (50 μg protein/mL) was added for 6 hours. After extensive washing, the adipocytes were incubated with or without palmitate (50 μmol/L) for 24 hours before measurement of *Saa3*, *Ccl2*, *Tnfa*, *Il1b*, *Il6*, *Bgn*, and *Vcan* gene expression. Data are representative of 3 independent experiments (*n* = 4) with mean ± SEM. *P < 0.001 vs. control HDL. **P < 0.001 vs. palmitate. ANOVA and Bonferroni’s post hoc test.

Silencing biglycan with a specific siRNA also restored the ability of SAA-enriched HDL from AgNO_3_-injected mice to inhibit macrophage inflammation (Figure 6). Collectively, these results suggest that biglycan at the cell surface of macrophages facilitates the trapping of SAA-enriched HDL and reduces the antiinflammatory properties of the lipoprotein by blocking its access to the plasma membrane, in a similar manner to what we have previously reported in fat cells (16).

## Discussion

In this study we demonstrate that HDL has antiinflammatory effects on both adipocytes and macrophages in vitro, which are lost when the HDL is enriched with SAA because of trapping of the SAA-containing HDL at the cell surface. The proteoglycan responsible for trapping SAA-enriched HDL at the surface of adipocytes is versican, whereas biglycan is responsible for trapping SAA-enriched HDL at the surface of macrophages. Interaction between SAA and these proteoglycans regulates HDL’s antiinflammatory properties on both adipocytes and macrophages. Similar to with adipocytes (16), the presence of SAA on the lipoprotein prevents HDL from removing cholesterol from LRs of palmitate-activated macrophages, as well as preventing the translocation of TLR4 and NOX2 to LRs, and reducing ROS generation.

SAA has a proteoglycan binding domain (18, 26). We previously have demonstrated that SAA-enriched HDL binds to chondroitin sulfate–rich proteoglycans on the surface of adipocytes (16). Elimination of either the SAA component in HDL or digesting the cell surface proteoglycan with chondroitin sulfate ABC lyase restored the antiinflammatory properties of HDL in adipocytes (16). This proteoglycan on adipocytes has subsequently been identified as versican (17). We now show that silencing versican eliminated the colocalization of SAA-HDL with the adipocyte cell surface, as well as blunting the ability of SAA-containing HDL to inhibit palmitate-induced inflammatory gene expression in the adipocytes. The crucial role of SAA in this interaction was confirmed by rendering control HDL dysfunctional by the addition of purified free SAA to the lipoprotein, which presumably displaced some APOA1 and became incorporated into the HDL (20).

We recently also have demonstrated that biglycan is the principal proteoglycan synthesized and secreted by adipose tissue macrophages in obesity (17). We now show that the presence of SAA on HDL leads to its colocalization with biglycan at the surface of macrophages, and blunting of the antiinflammatory properties of HDL in that cell type, similar to the effect of SAA-HDL in adipocytes. The essential role of biglycan was demonstrated by its silencing, which eliminated colocalization with SAA-enriched HDL at the macrophage surface and restored HDL’s antiinflammatory properties despite the presence of SAA on the lipoprotein. The mechanism by which HDL blunts inflammation appears to be similar in adipocytes and macrophages. Normal HDL prevents the palmitate-induced plasma membrane LR content, translocation of TLR4 and NOX2 to LRs, and ROS generation in macrophages, all of which were blunted by the presence of SAA on the lipoprotein, similar to our findings with adipocytes (16), although the form of NOX that is translocated in macrophages is NOX2 rather than NOX4 in adipocytes. As with fat cells, these effects were reversed when SAA was present in HDL, due to either the injection of AgNO_3_ to induce sterile inflammation or to the addition of purified SAA to HDL. Thus, it is now quite clear that blunting of the antiinflammatory properties of SAA-enriched HDL is in part regulated by the proteoglycans that associate with this complex.

Some investigators have reported that normal HDL has proinflammatory effects in macrophages after incubation of cells with high doses of HDL for 24 hours in the presence of LPS (24, 29). However, we did not observe a proinflammatory effect of control HDL in macrophages, likely due to difference in the concentration and/or duration of HDL exposure, in addition to the absence of LPS in our assay. Conversely, we clearly demonstrate antiinflammatory effects of control HDL on palmitate-induced chemokine and cytokine gene expression in macrophages, similar to our previous report in adipocytes (15, 16).

The major inducible forms of SAA present in HDL are SAA1.1 and SAA2.1 in mice and SAA2 and SAA1 in humans. They can be induced by acute inflammation, where plasma SAA levels can increase more than 100-fold (26), or more modestly (<10-fold) in chronic inflammatory states, such as obesity (15, 16, 30). These forms of SAA are made predominantly in the liver and are transported in plasma bound to HDL (26). Some SAA can be transported in lower density lipoproteins in obese mice (30, 31) and humans (32, 33). Our experiment used isolated HDL, which differs from plasma in which HDL coexists with lower density lipoproteins. It is possible that the presence of SAA in lower density lipoproteins, as can occur in subjects with diabetes and cardiovascular disease, may have additional effects on other cell types, particularly macrophages (33).

SAA4 is a constitutive form of SAA that is present at low levels in HDL in both mice and humans and is largely unaffected by inflammation (26), although it can increase modestly after LPS injection (28). SAA3 is a pseudogene in humans (26) but is produced in mice mainly by extrahepatic cells, such as adipocytes, macrophages, and enterocytes (26, 34), and is a chemotactic factor for inflammatory cells (35–37). We and others have shown that SAA3 can be transported in HDL and to a small extent in a free form in plasma after acute inflammation resulting from the injection of a high dose of LPS (27, 28) but not after more moderate chronic inflammation resulting from obesity (10). Whether SAA3 has proteoglycan-binding properties is not clear. To eliminate the possibility of SAA3 produced by macrophages being incorporated into the control HDL, we compared macrophages from WT and *Saa3^–/–^* mice. No differences were detected between either control or SAA-HDL exposed to either WT or *Saa3^–/–^* macrophages, suggesting that endogenous macrophage SAA3 does not contribute to inflammatory effects in this model.

In summary, control HDL has antiinflammatory effects in macrophages as well as in adipocytes. These antiinflammatory effects are blunted by the presence of SAA in HDL because of trapping of its SAA component by adipocyte-derived versican and macrophage-derived biglycan in adipose tissue. Findings from this study offer translational implications in which proteoglycans in adipose tissue could be a therapeutic target to modulate the function of HDL in obesity.

## Methods

### Animals, diets, and human subjects.

To generate control HDL or SAA-enriched HDL, 10-week-old female or male C57BL/6 mice were injected subcutaneously with AgNO_3_ (0.5 mL, 0.015 mg/mL) or PBS (38). After 24 hours, plasma was obtained for isolation of HDL by ultracentrifugation, as described previously (39, 40).

To investigate the immunohistochemical distribution of APOA1, SAA, macrophages, versican, and biglycan in adipose tissue in mice, we used tissues from a previous publication that showed immunostaining for MAC2, versican, and biglycan (17). For the present study, APOA1 and SAA were immunostained on adjacent sections. For the previous study (17), 10-week-old male C57BL/6 mice were fed either a chow (control) diet or an HFHS diet, which results in obesity, insulin resistance, adipose tissue, and systemic inflammation (30), for 16 weeks (*n* = 5–7 per group). At euthanasia, epididymal white adipose tissue (EWAT) was snap-frozen in liquid nitrogen and stored at −80°C or was fixed with 10% neutral-buffered formalin and embedded in paraffin as described previously (17).

To determine whether adipocyte-derived versican at the cell surface of adipocytes affects HDL’s antiinflammatory function, preadipocytes from adipocyte-specific versican *Adipoq-Cre/+ Vcan*^fl/fl^ (*Vcan^–/–^*) knockout mice were differentiated into mature adipocytes, as described previously (17).

To evaluate the relationship of APOA1 and SAA to the distribution of proteoglycans in human adipose tissue, we used adjacent sections from omental adipose tissue samples isolated from obese subjects undergoing gastric bypass surgery (18 women, 6 men, mean ± SD 45.4 ± 13.0 years) with BMI of 40 or greater (*n* = 24), for which we previously described the distributions of macrophages, versican, and biglycan (17). All tissues were obtained through the University of Washington Weight Loss Management Program and processed in the core laboratories of the University of Washington’s Nutrition Obesity Research Center. Samples were fixed with 10% neutral-buffered formalin and embedded in paraffin wax as described previously (17).

### Immunohistochemistry.

Adipose tissue, from mice or humans, fixed in 10% formalin for immunohistochemistry, were stained with antibodies against CD68 (Agilent, 1:200, M087601-2), MAC2 (R&D Systems, Bio-Techne, 1:25, AF1197), versican (MilliporeSigma, 1:250, AB1033), biglycan (Thermo Fisher Scientific, 1:200, PA5-76821), APOA1 (Rockland, 1:4000, 600-101-196), or SAA (R&D Systems, Bio-Techne, 1:25, AF2948) and photographed as described previously (41). Area quantification for each staining was performed on digital images of immunostained tissue sections using Image Pro Plus 6.0 (Media Cybernetics).

### Cell culture.

We propagated and differentiated 3T3-L1 murine preadipocytes (ATCC) according to standard procedures (42, 43), except that the medium was changed daily. For some experiments, preadipocytes isolated from the stromal vascular fraction of collagenase-digested EWAT from 10-week-old male *Adipoq-Cre/+ Vcan*^fl/fl^ (*Vcan^–/–^*) and *Adipoq-Cre/+ Vcan*^+/+^ (*Vcan^+/+^*) mice (17) were grown to confluence and differentiated in DMEM (Hyclone) containing 32 μmol/L dexamethasone (MilliporeSigma), 780 μmol/L 3-isobutyl-1-methylxanthine (MilliporeSigma), 10 μg/mL bovine insulin (MilliporeSigma), 1 μmol/L rosiglitazone (Cayman Chemical), and 1 μmol/L indomethacin (MilliporeSigma) for 4 days. Differentiated adipocytes were cultured for 7 days with daily replenishment of DMEM.

TG-elicited peritoneal macrophages were isolated 4 days after the intraperitoneal injection of WT C57BL/6 or SAA3-knockout (*Saa3^–/–^*) mice with TG (BD). To remove nonadherent peritoneal cells, the plates were washed extensively after adherence of macrophages to the plates for 60 minutes.

BMDMs were isolated from the tibias and fibulas of C57BL/6 mice, then pooled and plated on 6-well plates in RPMI 1640 medium (Hyclone) containing 10% FBS (Hyclone) and 30% L929 conditioned medium (44). Medium was replaced on days 2 and 4 (with retention of floating and attached cells) and on day 6, when floating cells were discarded. To induce M1 macrophages, BMDMs were stimulated for 24 hours on day 7 with IFN-γ (10 ng/mL; R&D Systems, Bio-Techne) and LPS (1 ng/mL; Cayman Chemical). For M2 macrophages, BMDMs were stimulated for 48 hours with IL-4 (10 ng/mL; R&D Systems, Bio-Techne).

Inflammation was induced in both adipocytes and macrophages by incubation with palmitate (16:0) (MilliporeSigma), conjugated with albumin as described previously (43). Briefly, palmitate was first dissolved in NaOH (100 mmol/L) and conjugated with fatty acid–free albumin (MilliporeSigma) at a molar ratio of 3:1 (palmitate/albumin). Fully differentiated adipocytes and macrophages were pretreated with 50 μg protein/mL of HDL for 6 hours, then washed 3 times with PBS. Adipocytes and macrophages then were incubated for 24 hours with 250 μmol/L palmitate for measurement of gene expression by real-time quantitative reverse transcription PCR (RT-PCR), as described previously (15, 16).

### In vitro versican and biglycan gene silencing.

For experiments in which we tested the roles of versican and biglycan on HDL’s antiinflammatory function, 3T3-L1 adipocytes and TG-elicited peritoneal macrophages were transiently transfected with siRNA duplexes for versican and biglycan, respectively, or scrambled sequences, synthesized and purified by Ambion using the DeliverX system (Panomics), as described previously (43). Versican and biglycan expression levels were markedly silenced compared with transfection with control scrambled constructs and untreated cells (>90% reduction).

### Immunofluorescence.

To evaluate the localization of HDL and the ECM in 3T3-L1 adipocytes, and TG-elicited macrophages, cells were cultured on glass coverslips. The various HDL preparations were added for 6 hours, then fixed in 2% formalin for 5 minutes. The ECM of adipocytes and macrophages was stained using versican and biglycan antibodies followed by an Alexa Fluor 594–conjugated secondary antibody (Molecular Probes, Thermo Fisher Scientific). For HDL and SAA staining, cells were stained with APOA1 (Rockland, 1:500, 600-101-196) or SAA (R&D Systems, Bio-Techne, 1:500, AF2948) antibodies, followed by FITC-conjugated secondary antibody (Molecular Probes, Thermo Fisher Scientific), as described previously (16). For some experiments, versican and biglycan were silenced using specific siRNAs (Ambion) before the addition of HDL. Nuclei were counterstained with DAPI. Cells were photographed using a Nikon Eclipse 80i fluorescence microscope.

### Real-time quantitative reverse transcription polymerase chain reaction.

RNA isolated from samples (2 μg total) was reverse-transcribed into cDNA. RT-PCR was performed using the TaqMan Master kit (Thermo Fisher Scientific) in the ABI prism 7900HT system (30, 45). Murine *Saa3*, *Ccl2*, *Tnfa*, *Il1b*, *Il6*, *Vcan*, *Bgn*, *Arg1*, *Nos2*, *Cd206*, and *Gapdh* primers with FAM probes were obtained from Applied Biosystems (Assay-on-Demand, Thermo Fisher Scientific). Each sample was analyzed in triplicate and normalized using *Gapdh* as control.

### LR measurement.

LRs in plasma membranes of TG-elicited peritoneal macrophages were quantified as described previously (46) using Alexa Fluor 594–conjugated cholera toxin β (CTB, Molecular Probes, Thermo Fisher Scientific). Briefly, cultured 3T3-L1 adipocytes were incubated with 1 μg/mL of Alexa Fluor 594–conjugated CTB for 15 minutes at 4°C. After washing twice with cold PBS, cells were fixed in 4% paraformaldehyde for 20 minutes at 4°C. CTB staining of fixed cells was analyzed by FACS (FACS RUO, BD) as a measure of LR content (15).

### Quantification of ROS.

ROS generation by adipocytes was assessed as CM-H_2_DCFDA (Molecular Probes, Thermo Fisher Scientific) fluorescence monitored by FACS (FACS RUO, BD), as described previously (43, 46).

### Detergent-free LR fractionation.

LR and non-LR fractions from adipocytes and macrophages were obtained by Optiprep gradient centrifugation using a detergent-free protocol, as described previously (46). Briefly, cell pellets were homogenized in buffer (250 mmol/L sucrose, 1 mmol/L EDTA, 500 mmol/L sodium bicarbonate, pH 11). After centrifugation (1000*g*, 10 minutes), the postnuclear supernatant fraction was added to 60% Optiprep to a final concentration of 35% Optiprep, overlaid on a discontinuous gradient of 5%–35% Optiprep, and centrifuged at 326,512*g* in a Beckman NVT 65.2 rotor for 90 minutes at 4°C. Nine 0.5 mL fractions were collected and subjected to immunoblotting using antibodies against TLR4 (Cell Signaling Technology, 1:1000, 14358), NOX2 (Invitrogen, Thermo Fisher Scientific, 1:1000, PA5-76034), and caveolin-1 (Cell Signaling Technology, 1:1000, 3238).

### Statistics.

Statistical significance was determined with SPSS (Windows version 19) or OriginPro software (version 8.6; Origin Laboratory). All data are shown as mean ± SEM. Student’s *t* test was used to detect differences within groups when applicable (2 tailed). One-way ANOVA was used to compare differences among all groups, and Bonferroni’s post hoc testing was used to detect differences among mean values of the groups. A *P* value less than 0.05 was considered statistically significant.

### Study approval.

All experimental procedures in mice were undertaken with approval from the Institutional Animal Care and Use Committee of the University of Washington (protocols 3104-01 and 4237-01). All human subjects provided written informed consent and authorization for tissue biopsy (protocol 00002737).

## Author contributions

CYH, IK, MO, TW, and SW conducted the experiments. All authors interpreted the data and assisted with editing the manuscript. CYH, TNW, and AC designed the experiments, supervised the work, interpreted the data, and wrote the manuscript.

## Supplementary Material

supplemental data

## Version 1 09/24/2020

In-Press Preview

## Version 2 10/15/2020

Electronic publication
