# Supplementary material for: Serum amyloid A–containing HDL binds adipocyte-derived versican and macrophage-derived biglycan, reducing its antiinflammatory properties
Source: JCI Insight. 2020 Sep 24;5(20):e142635. doi: 10.1172/jci.insight.142635 (PMC7605543; doi:10.1172/jci.insight.142635)
Supplement: supplemental data [file jciinsight-5-142635-s096.pdf]

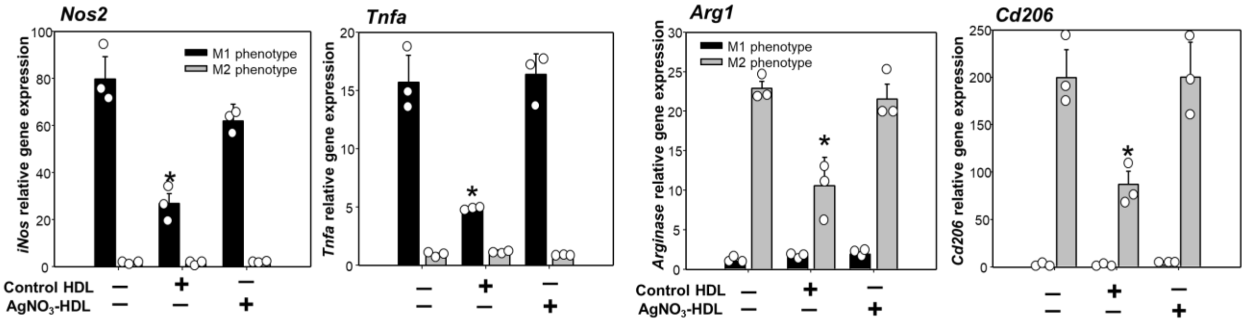

Supplemental Figure I. Control HDL blocks the differentiation of bone marrow-derived macrophages into M1 and M2 type, while HDL isolated from $AgNO_3$-injected mice does not. Bone marrow-derived macrophages were isolated and pre-exposed with HDL from PBS- or $AgNO_3$-injected C57BL/6 mice for 6 hours prior to differentiation to M1 or M2 types as described in methods. After differentiation, *Nos2* and *Tnfa* gene expression (**A**), markers for M1 type, and *Arg1* and *Cd206* gene expression (**B**), markers for M2, were measured by RT-PCR. Data are representative of 3 independent experiments (n=3) with mean ± SEM. *$p < 0.001$ vs. $AgNO_3$-HDL. ANOVA and Bonferroni post-hoc test.
